# Small non‐coding RNA signatures in atrial appendages of patients with atrial fibrillation

**DOI:** 10.1111/jcmm.18483

**Published:** 2024-06-22

**Authors:** Yuhong Zeng, Zhiquan Yuan, Jun Li, Lanqing Yang, Chengying Li, Ying Xiang, Long Wu, Tingting Xia, Li Zhong, Yafei Li, Na Wu

**Affiliations:** ^1^ Department of Epidemiology, College of Preventive Medicine Army Medical University (Third Military Medical University) Chongqing People's Republic of China; ^2^ Thoracic and Cardiac Surgery, Southwest Hospital The First Affiliated Hospital of Army Medical University (Third Military Medical University) Chongqing People's Republic of China; ^3^ Cardiovascular Disease Center Third Affiliated Hospital of Chongqing Medical University Chongqing People's Republic of China

**Keywords:** atrial fibrillation, ribosomal RNA‐derived small RNAs, small non‐coding RNAs, small nucleolar RNAs, transfer RNA‐derived small RNAs

## Abstract

The development of high‐throughput technologies has enhanced our understanding of small non‐coding RNAs (sncRNAs) and their crucial roles in various diseases, including atrial fibrillation (AF). This study aimed to systematically delineate sncRNA profiles in AF patients. PANDORA‐sequencing was used to examine the sncRNA profiles of atrial appendage tissues from AF and non‐AF patients. Differentially expressed sncRNAs were identified using the R package DEGseq 2 with a fold change >2 and *p* < 0.05. The target genes of the differentially expressed sncRNAs were predicted using MiRanda and RNAhybrid. Gene Ontology (GO) categories and Kyoto Encyclopedia of Genes and Genomes (KEGG) pathway analyses were performed. In AF patients, the most abundant sncRNAs were ribosomal RNA‐derived small RNAs (rsRNAs), followed by transfer RNA‐derived small RNAs (tsRNAs), and microRNAs (miRNAs). Compared with non‐AF patients, 656 rsRNAs, 45 miRNAs, 191 tsRNAs and 51 small nucleolar RNAs (snoRNAs) were differentially expressed in AF patients, whereas no significantly differentially expressed piwi‐interacting RNAs were identified. Two out of three tsRNAs were confirmed to be upregulated in AF patients by quantitative reverse transcriptase polymerase chain reaction, and higher plasma levels of tsRNA 5006c‐LysCTT were associated with a 2.55‐fold increased risk of all‐cause death in AF patients (hazard ratio: 2.55; 95% confidence interval, 1.56–4.17; *p* < 0.001). Combined with our previous transcriptome sequencing results, 32 miRNA, 31 snoRNA, 110 nucleus‐encoded tsRNA, and 33 mitochondria‐encoded tsRNA target genes were dysregulated in AF patients. GO and KEGG analyses revealed enrichment of differentially expressed sncRNA target genes in AF‐related pathways, including the ‘calcium signaling pathway’ and ‘adrenergic signaling in cardiomyocytes.’ The dysregulated sncRNA profiles in AF patients suggest their potential regulatory roles in AF pathogenesis. Further research is needed to investigate the specific mechanisms of sncRNAs in the development of AF and to explore potential biomarkers for AF treatment and prognosis.

## 1 INTRODUCTION

Atrial fibrillation (AF), the most prevalent cardiac arrythmia in clinical practice, affects over 30 million individuals worldwide.^1^ The prevalence of AF increases with age, ranging from approximately 3% in the general population to 8%–10% in older adults.^2^, ^3^ AF is associated with increased risks of mortality and other adverse cardiovascular events such as heart failure, stroke and myocardial infarction.^4^ Current therapies for AF mainly focus on symptom improvement, thromboembolism and cardiomyopathy prevention,^5^ but their efficacy is suboptimal, with a high recurrence rate, poor tolerance and possible adverse side effects.^6^ Therefore, further exploration of the pathophysiological mechanisms that contribute to AF may help improve treatment outcomes.

Small non‐coding RNAs (sncRNAs), such as microRNAs (miRNAs), PIWI‐interacting RNAs (piRNAs), transfer RNA‐derived small RNAs (tsRNAs), small nucleolar RNAs (snoRNAs), are a subclass of RNAs with less than 200 nucleotides in length and lack protein‐coding potential.^7^ The sncRNAs were once considered ‘evolutionary junk’; however, high‐throughput sequencing technologies have enabled our understanding of the potential role of sncRNAs in several diseases, including AF.^8^, ^9^ Among all the sncRNAs, miRNAs, which play important roles in complex human diseases through post‐transcriptional regulation of gene expression, have been extensively investigated.^10^ Their expression and underlying mechanisms in various diseases have been previously reported.^10^, ^11^, ^12^ Comprehensive miRNA databases have been established, and various computational models for predicting miRNA‐disease associations have been developed.^11^, ^12^ Moreover, miRNAs are involved in structural remodelling, electrical remodelling and autonomic remodelling in AF.^9^ However, the roles of other sncRNAs in AF remain poorly explored. To our knowledge, only two studies have explored the role of tsRNAs in AF.^13^, ^14^ One study examined the tsRNA expression profiles in three pairs of AF and control heart tissues using high‐throughput sequencing.^13^ Another study found that tsRNA‐5008a was highly expressed in an AF mouse model and promoted myocardial fibrosis by targeting SLC7A11.^14^ Other sncRNAs, such as snoRNAs or piRNAs, have not been studied in AF. Furthermore, no study has systematically explored the expression signatures of the different classes of sncRNAs in AF to date. Given that other sncRNAs have been implicated in diverse regulatory functions such as reverse transcription, post‐transcriptional gene silencing, ribosome biogenesis and epigenetic modification, and considering their roles in other diseases,^15^, ^16^, ^17^, ^18^ delineating the sncRNA expression profiles of AF (not only the expression profile of a class of sncRNAs) and exploring their potential roles in AF biology is warranted.

Revealing small RNA profiles may indicate the classes of sncRNAs that are dominant in AF pathogenesis, and differentially expressed sncRNA profiles further lay the foundation for basic research. Therefore, this study aimed to examine the expression profiles of sncRNAs in atrial appendage tissues from AF patients and non‐AF patients and identify differentially expressed sncRNAs. Our study helps to better understand the regulatory role of sncRNAs in AF and provides a basis for further exploration of the specific mechanism by which sncRNAs affect the occurrence and development of AF and the potential use of sncRNAs as biomarkers for AF treatment and prognosis.

## 2 MATERIALS AND METHODS

The flowchart of data collection and method implementation is illustrated in Figure S1.

### 2.1 Patients and sample collection

Atrial appendage tissues were collected from patients with valvular heart disease with newly diagnosed AF and those without AF during valve replacement or valve repair surgery at Southwest Hospital, Army Medical University, Chongqing, China. Atrial tissues were frozen in liquid nitrogen immediately after surgical resection. Patients diagnosed with other cardiac arrhythmias, malignant tumours, hyperthyroidism, hypothyroidism or other cardiovascular diseases, such as coronary heart disease, congenital heart disease, or heart failure, were excluded. Patients without AF were matched in a 1:1 ratio to AF patients based on age (±1 year) and sex. Clinical characteristics of the patients were extracted from electronic medical records using a pre‐designed form, which included patient demographics, lifestyle, medical history and physical examination findings.

Plasma was collected from patients with non‐valvular AF in a retrospective cohort. Patients diagnosed with structural heart disease, moderate‐to‐severe mitral stenosis, prosthetic valve replacement, other cardiac arrhythmias or cardiovascular disease, malignant tumours, or hyperthyroidism or hypothyroidism were excluded. The venous blood of non‐valvular AF patients who had not undergone any treatment within 48 h after admission was collected, and the upper plasma was aspirated after centrifugation and stored in the refrigerator at −80*°*C. Clinical characteristics of patients and outcome data, including death, were collected using pre‐designed forms. Trained investigators followed up on death information through the telephone and documented the cause and time of death.

The Ethics Committee of Southwest Hospital, Army Medical University, granted ethical approval for the study, and written informed consent was obtained from all participants.

### 2.2 Small RNA library construction and sequencing

Total RNA was isolated from the atrial appendages of patients using TRIzol reagent (1 mL; Invitrogen; 15,596,018) according to the manufacturer's instructions. The concentration of RNA was measured using a Qubit fluorometer, and the purity and integrity of the RNA were analysed using a Qsep100 automatic nucleic acid protein analyser. The total RNA concentration ranged between 121.6 and 980 ng/μL, and all RNAs were qualified for further sequencing.

Small RNA library construction and sequencing were performed by Guangzhou Epibiotek Co., Ltd., China, according to the PANDORA‐seq (Panoramic RNA Display by Overcoming RNA Modification Aborted Sequencing) protocol.^19^ PANDORA‐seq is a new sequencing technology that removes key RNA modifications that may block adapter ligation and reverse transcription.^19^ In brief, the RNA was first treated sequentially with T4 polynucleotide kinase (T4PNK) and dealkylating enzyme α‐ketoglutarate‐dependent hydroxylase (AlkB, Epibiotek, R1822). The RNA segment was then separated using polyacrylamide gel electrophoresis (PAGE), and a 15‐ to 45‐nucleotide stripe was selected and recycled. The 3′ and 5′ adapter were obtained from the QIAseq® miRNA Library Kit (QIAGEN: 331505) and ligated sequentially. After PCR amplification, the product was purified from the PAGE gel, and the qualified libraries were amplified on cBot to generate clusters on the flow cell. Finally, the amplified flow cell was sequenced using the SE75 strategy on an Illumina system.^19^

Clean sequencing reads were annotated using SPORTS1.1 software^20^ with one mismatch tolerance, and were sequentially aligned to the miRNA database miRBase 21,^21^ genomic tRNA database GtRNAdb,^22^ mitochondrial tRNA database mitotRNAdb,^23^ and rRNA and YRNA databases assembled from the National Center for Biotechnology Information nucleotide and gene database, piRBase^24^ and piRNABank,^25^ and the non‐coding RNAs defined by Ensembl^26^ and Rfam 12.3.^27^ The nomenclature of tsRNA was also referred to the authoritative database tRFdb.^28^

### 2.3 Differentially expressed sncRNA analysis and target gene prediction

The R package DEGseq 2 was used to identify the differentially expressed sncRNAs.^29^ Significant differential expression was determined by a fold change in normalized reads of the exon model per million mapped reads (RPM) >2 and a significance level of *p* < 0.05. The targeted regulation of differentially expressed sncRNAs was predicted using MiRanda (https://www.mirandalawfirm.com/en) and RNAhybrid,^30^ and the results were intersected to generate the final target genes.

### 2.4 Gene Ontology and Kyoto Encyclopedia of Genes and Genomes pathway analysis

Gene Ontology (GO) terms and Kyoto Encyclopedia of Genes and Genomes (KEGG) pathway analyses were performed to determine the biological implications and metabolic pathways of the target genes of differentially expressed sncRNAs. Fisher's exact test was used to identify significant GO categories and pathways, and the threshold value for significance was set at *p* < 0.05.

### 2.5 Validation by quantitative reverse transcriptase polymerase chain reaction (qRT‐PCR)

Three differentially expressed tsRNAs and three snoRNAs detected by sequencing were selected for further validation by qRT‐PCR in expanded atrial tissue samples. The expression of one tsRNA in the plasma of patients with non‐valvular AF was analysed to examine its correlation with all‐cause mortality. Total RNA was extracted from the atrial tissues using the TRIzol reagent (Invitrogen; 15,596,018). QIAzol (Qiagen, Germany) and the miRNeasy Serum/Plasma Kit (50) (Qiagen, Germany) were used to extract total RNA from the plasma. Reverse transcription of tsRNAs and snoRNAs was performed using miRNAFirst Strand cDNA Synthesis kit (Stem‐loop method) (Sangon Biotech, China) and PrimeScript RT Reagent kit (Takara, Shiga, Japan), respectively. SYBR Premix Ex Taq (Takara) was used for the qPCR analysis. The relative expression of tsRNAs and snoRNAs was normalized to U6, and the fold change was calculated using the 2^−△△Ct^ method. Human cardiac fibroblasts (Xinrun Biotech Co., Ltd., Wuxi, China) were used as an interplate control to measure tsRNA expression in plasma samples. Primer sequences are listed in Table S1.

### 2.6 Statistical analysis

Continuous variables with normal and skewed distributions are presented as means ± standard deviations and medians and interquartile ranges, respectively. The *t*‐test, or Mann–Whitney *U*‐test, was used to assess the statistical significance of differences in means or medians between the groups. Categorical variables are expressed as counts and proportions, and the chi‐square test or Fisher's exact test was used to compare differences between the groups when appropriate. The optimal cutoff value for the plasma level of tsRNA 5006c‐LysCTT was determined using the surv_cutpoint function. The multivariable Cox proportional hazards model was used to analyse the association between the plasma levels of tsRNA 5006c‐LysCTT and all‐cause death by adjusting for potential confounding variables with *p* < 0.1 in the univariable Cox proportional hazards model. Two‐sided *p* values <0.05 were considered statistically significant. All analyses were performed using R 4.3.3 (R Core Team, Vienna, Austria) and GraphPad Prism 9.5 (La Jolla, CA, USA).

## 3 RESULTS

### 3.1 sncRNAs profiles in AF patients and non‐AF patients

In total, atrial appendages from six AF patients and six non‐AF patients were used to analyse sncRNA profiles, and their clinical characteristics are shown in Table 1. No significant differences in age, body mass index, smoking status, or other characteristics were observed between the two groups.

**TABLE 1:** Clinical characteristics of patients with AF and without AF.

Characteristic	AF patients (*n* = 6)	Non‐AF patients (*n* = 6)	*p* value
Age, mean ± SD	49.7 ± 6.7	49.5 ± 6.2	0.965
Male, *n* (%)	3 (50.0)	3 (50.0)	>0.1
BMI, mean ± SD	24.9 ± 4.8	22.9 ± 3.1	0.425
Smoking status, *n* (%)			
Current	2 (33.3)	2 (33.3)	>0.1
Never	3 (50.0)	3 (50.0)	
Ever	1 (16.7)	1 (16.7)	
Drinking status, *n* (%)			
Current	0 (0)	0 (0)	>0.1
Never	4 (66.7)	5 (83.3)	
Ever	2 (33.3)	1 (16.7)	
Hypertension, *n* (%)	0 (0)	0 (0)	>0.1
Diabetes, *n* (%)	0 (0)	0 (0)	>0.1
NYHA classification, *n* (%)			
I	1 (16.7)	0 (0)	>0.1
II	1 (16.7)	2 (33.3)	
III–IV	4 (66.7)	4 (66.7)	

**FIGURE 1:**
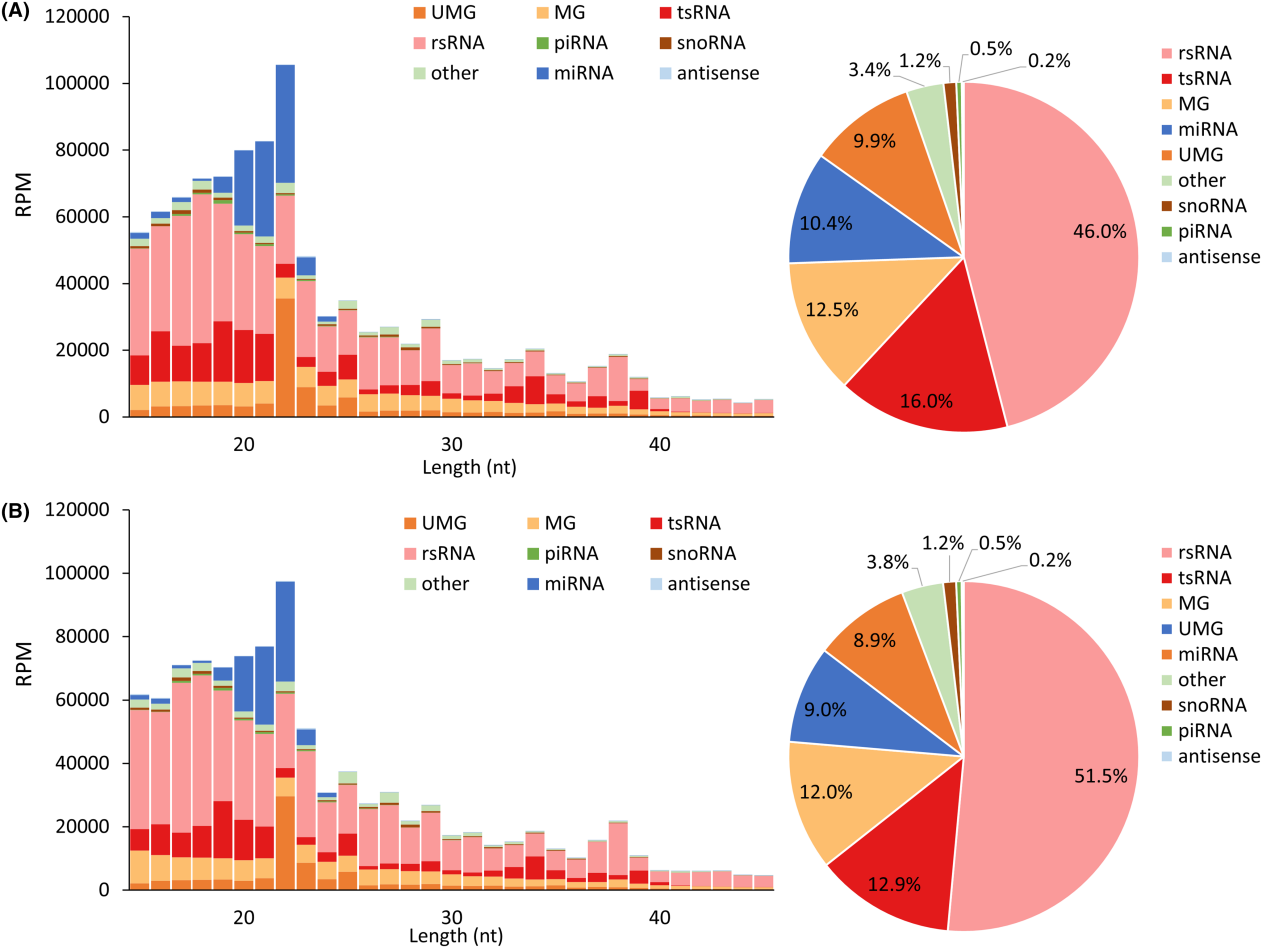
Length distribution and relative abundance of small noncoding RNAs (sncRNAs) types in atrial appendages of (A) atrial fibrillation (AF) patients and (B) non‐AF patients. (UMG, unmatched genome; MG, matched genome but lack annotation information; RPM, reads of exon model per million mapped reads).

**FIGURE 2:**
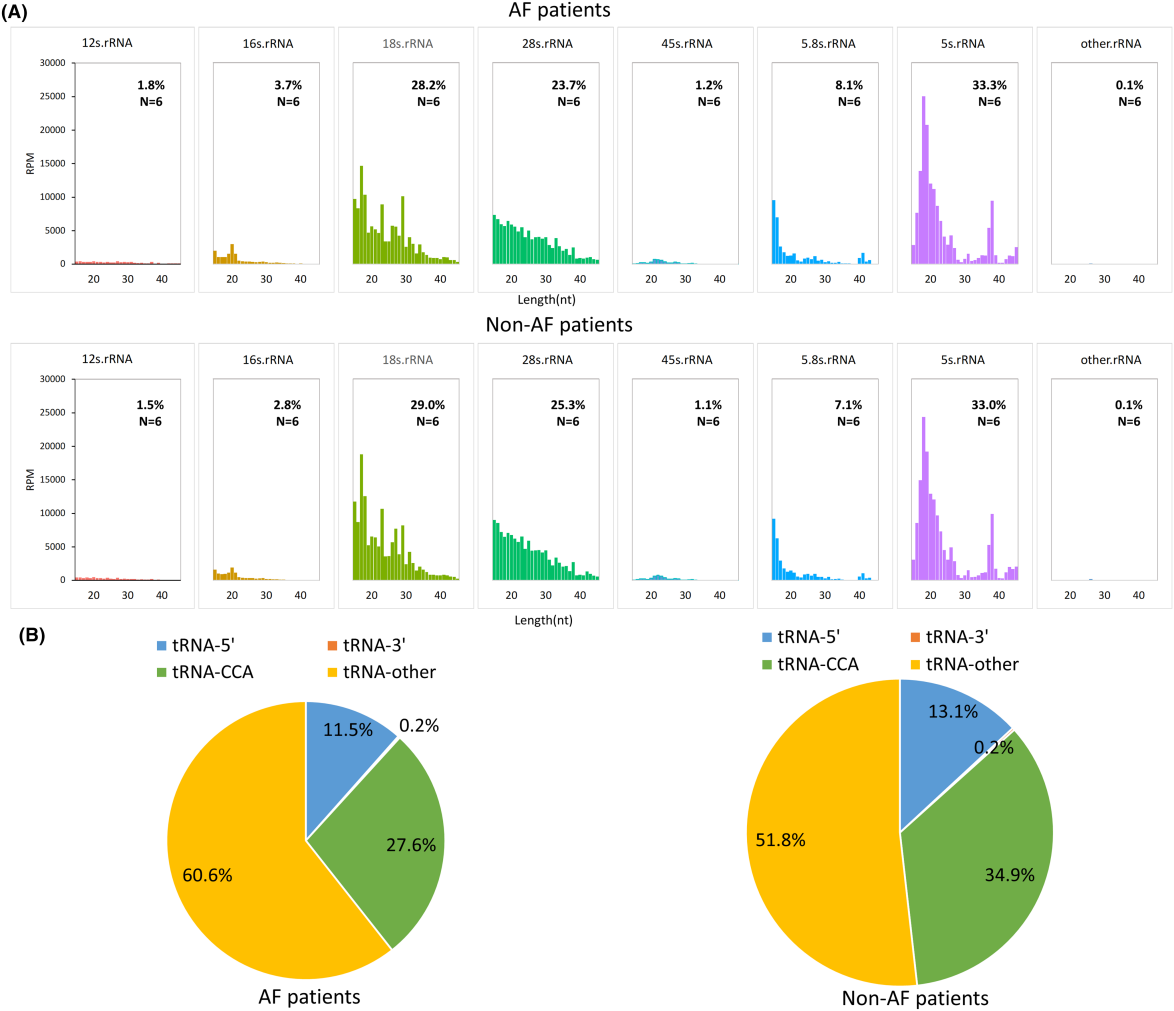
Landscape of ribosomal RNA‐derived small RNAs (rsRNAs) and transfer RNA‐derived small RNAs (tsRNAs) in AF patients and non‐AF patients. (A) Length distribution of different type of rsRNAs. (B) Relative proportion of different types of tsRNAs.

Among all sncRNAs, ribosomal RNA‐derived small RNAs (rsRNAs) were the most abundant in both AF (46.0%) and non‐AF patients (51.5%), followed by tsRNAs (AF, 16.0%, non‐AF, 12.9%) and miRNAs (AF, 10.4%; non‐AF, 8.9%) (Figure 1). Most rsRNAs were derived from 5 s ribosomal RNA (rRNA), 18 s rRNA, and 28 s rRNA, which together accounted for 85.2% and 87.3% of rsRNAs in AF and non‐AF patients, respectively (Figure 2A). The tsRNAs were classified into four categories, which included 5′ tsRNA (derived from the 5′ end of pre−/mature tRNA), 3′ tsRNA (derived from the 3′ end of pre‐tRNA), 3′ tsRNA‐CCA (derived from the 3′ end of mature tRNA), and other tsRNA. Our findings indicated that 3′ tsRNA‐CCA and other tsRNAs accounted for > 80% of tsRNAs in AF and non‐AF patients (Figure 2B).

### 3.2 Differentially expressed sncRNAs in AF relative to non‐AF

**FIGURE 3:**
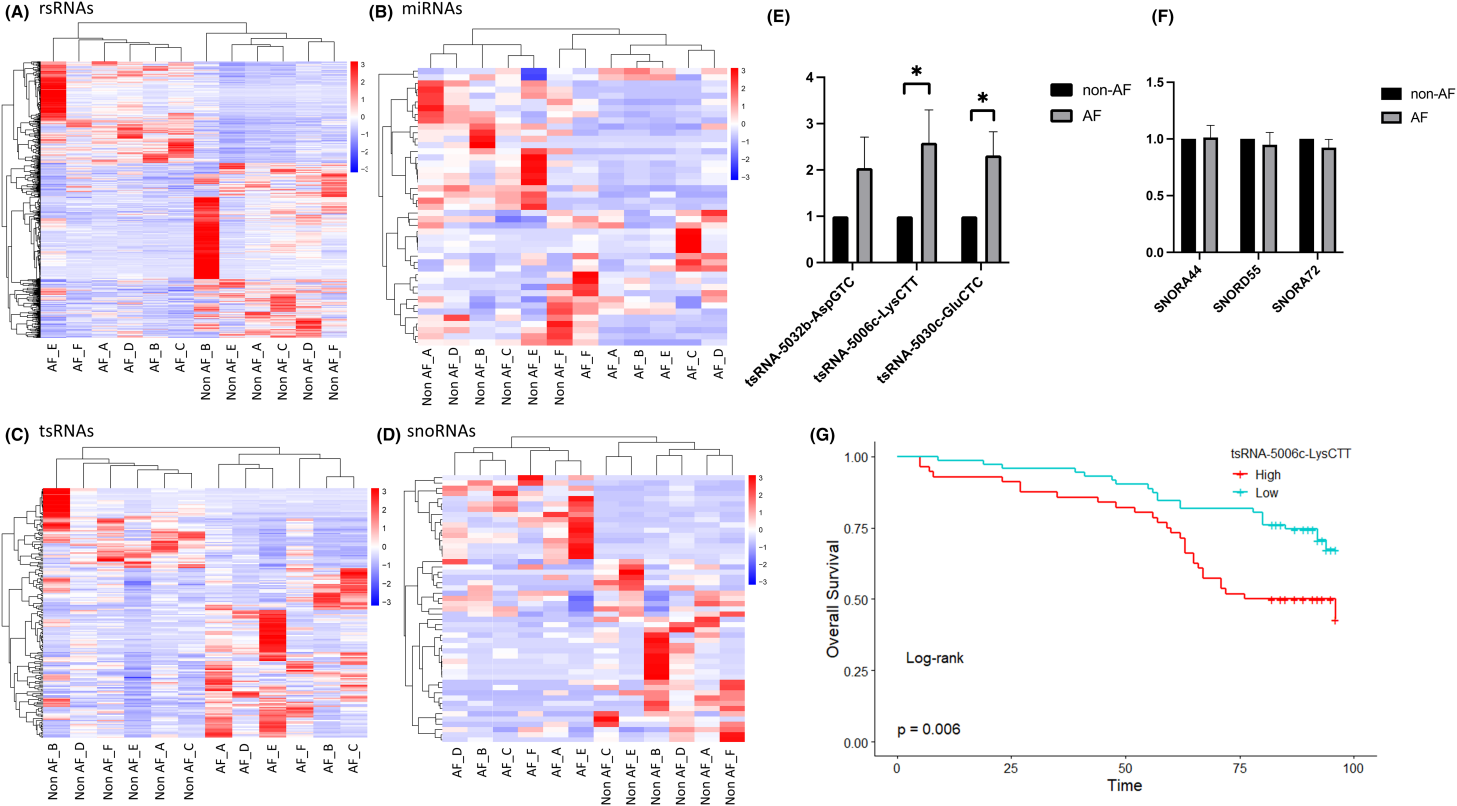
Comparison of differentially expressed sncRNAs between AF and non‐AF patients. (A–D) Heatmaps of the differentially expressed rsRNAs, microRNAs (miRNAs), tsRNAs and small nucleolar RNAs (snoRNAs). (E, F) Quantitative reverse transcriptase polymerase chain reaction analysis of the expression of three randomly selected differentially expressed tsRNAs (*n* = 25 pairs) and snoRNAs (*n* = 17 pairs) relative to U6 in patients with and without AF. (G) Kaplan–Meier curves for all‐cause death between AF patients with high and low levels of tsRNA‐5006c‐LysCTT. *p < 0.05 versus corresponding non‐AF group.

For rsRNAs, there were 656 differentially expressed fragments between AF and non‐AF patients, of which > 50% were derived from 28 s‐rRNA and approximately 25% were derived from 18 s‐rRNA. Compared with non‐AF patients, 45 differentially expressed miRNA sequences were identified in AF patients, of which 19 were upregulated and 26 were downregulated. For tsRNAs and snoRNAs, 191 and 51 differentially expressed fragments were discovered, respectively, whereas no differentially expressed fragments were found for piRNAs. Figure 3A–D shows the comparison of heatmaps and hierarchical clustering of differentially expressed rsRNAs, miRNAs, tsRNAs and snoRNAs between AF and non‐AF patients. Among the 191 differentially expressed tsRNAs, 104 were mitochondria‐encoded tsRNAs, 86 were nucleus‐encoded tsRNAs, and only 1 was from pre‐tRNA‐Arg‐TCT. The top 10 upregulated and downregulated differentially expressed sequences of rsRNAs, miRNAs, tsRNAs and snoRNAs are listed in Tables S2–S5.

Three tsRNAs and three snoRNAs were selected for further validation by qRT‐PCR in extended atrial appendage samples (Figure 3E,F). Consistent with the sequencing results, compared with non‐AF patients, two tsRNAs (tsRNA‐5006c‐LysCTT and tsRNA‐5030c‐GluCTC) were significantly upregulated in AF patients, and the remaining one showed an increasing trend but did not reach statistical significance. Although the qRT‐PCR results of the two selected snoRNAs were consistent with the sequencing results, the expression of all three selected snoRNAs did not differ significantly in AF patients.

### 3.3 tsRNA 5006c‐LysCTT was associated with an increased risk of death in AF patients

To further explore the role of differentially expressed sncRNAs as biomarkers in AF prognosis, the most highly expressed tsRNA, 5006c‐LysCTT, identified by qRT‐PCR in the extended validation samples was selected to examine its association with all‐cause death in AF patients. A total of 127 AF patients were enrolled, with a median follow‐up time of 89 months; their clinical characteristics are summarized in Table S6. During the follow‐up period, mortality was reported in 50 patients, with a mortality rate of 6.12/100 person‐years. Compared with patients with low plasma tsRNA 5006c‐LysCTT levels, patients with high tsRNA 5006c‐LysCTT levels had higher mortality rates (Figure 3G). After adjusting for age, education level, and CHA_2_DS_2_‐VASc score, high tsRNA 5006c‐LysCTT was associated with a 2.55‐fold increased risk of all‐cause death (hazard ratio [HR]: 2.55; 95% confidence interval [CI], 1.56–4.17; *p* < 0.001), which suggested that tsRNA 5006c‐LysCTT may be a potential biomarker for AF prognosis.

### 3.4 Target gene prediction and GO and KEGG pathway analysis

To explore the potential biological functions of the differentially expressed miRNAs, tsRNAs, and snoRNAs, MiRanda and RNAhybrid were used to predict their target genes. The results revealed 1409 and 1940 common target genes for all differentially expressed miRNAs and snoRNAs, respectively. Additionally, 14,323 common target genes were predicted for all differentially expressed nucleus‐encoded tsRNAs and 2486 common target genes for mitochondria‐encoded tsRNAs (2474 for mitochondria‐encoded tsRNAs without 3′ end CCA, 12 for mitochondria‐encoded tsRNAs with 3′ end CCA).

To further delineate the target genes associated with AF, the predicted target genes of the differentially expressed sncRNAs were intercrossed with our previous transcriptome sequencing results from AF and non‐AF atrial tissues.^31^ Finally, abnormal expression of 32 miRNA, 31 snoRNA, 110 nucleus‐encoded tsRNA, and 33 mitochondria‐encoded tsRNA target genes was identified in AF patients. GO analysis was performed on the target genes of the corresponding sncRNAs, and the top 10 enriched biological processes, cell components, and molecular functions are listed in Figure S2. Regarding biological processes, the target mRNAs of the differentially expressed sncRNAs were mainly involved in the ‘regulation of blood vessel endothelial cell migration,’ ‘action potential,’ ‘positive regulation of calcium ion transport,’ and ‘negative regulation of voltage‐gated calcium channel activity.’ Regarding molecular functions, the target genes were enriched in ‘transforming growth factor beta receptor activity,’ ‘voltage‐gated monoatomic cation channel activity,’ ‘neurotransmitter transmembrane transporter activity,’ and ‘transmembrane transporter binding.’

**FIGURE 4:**
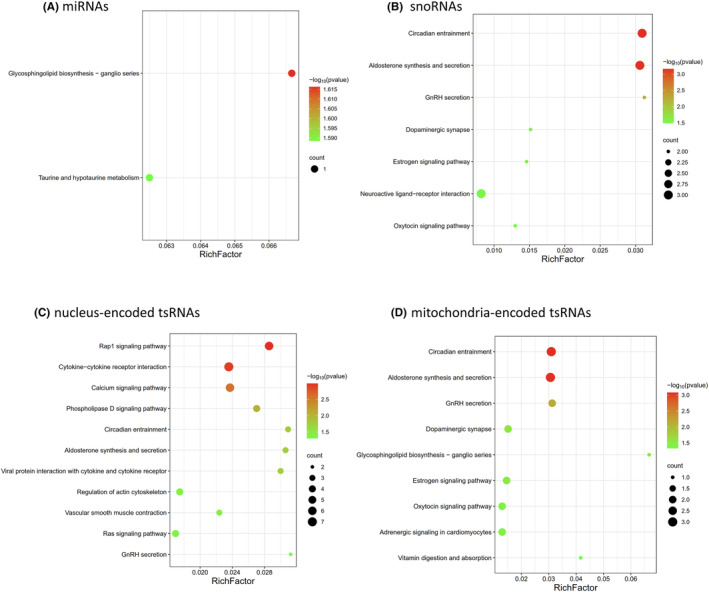
Pathway analyses of target genes of differently expressed (A) miRNAs, (B) snoRNAs (C) nucleus‐encoded tsRNAs and (D) mitochondria‐encoded tsRNAs.

Additionally, KEGG pathway analyses demonstrated that the presumed target genes of sncRNAs were significantly enriched in ‘calcium signaling pathway’ and ‘adrenergic signaling in cardiomyocytes,’ suggesting the potential functional roles of differentially expressed sncRNAs in AF development (Figure 4).

## 4 DISCUSSION

This study was the first to comprehensively characterize sncRNA profiles in atrial appendages from AF and non‐AF patients, reveal differentially expressed sncRNAs specific to AF, and delineate the target gene profiles and pathways associated with differentially expressed sncRNAs. Our study findings facilitate further exploration of the mechanism of action of sncRNAs in AF and identify new potential therapeutic targets and biomarkers.

Although several miRNAs have predictive roles in AF prognosis and are associated with structural, electrical and autonomic neural remodelling in AF,^9^ and miRNA‐based therapies are also expected to be translated from the research setting to clinical applications,^8^ the potential of other sncRNAs in the disease has been underestimated and poorly studied owing to low detection rates. Conventional high‐throughput sequencing builds complementary DNA libraries by adapter ligation of the 3′‐ and 5′‐ends of RNA, which may be blocked when sncRNAs have specific 3′‐ or 5′‐end modifications.^32^ Additionally, RNA methylation may disrupt reverse transcription.^19^, ^33^, ^34^ Therefore, sncRNAs with large modifications may not be identified using conventional high‐throughput sequencing. Compared to conventional high‐throughput sequencing, PANDORA sequencing with AlkB and T4PNK enzyme treatment resulted in a significant increase in reads for tsRNAs and rsRNAs without significant alterations in miRNA profiles, which revealed a broader and more accurate composition of sncRNAs in a wide range of mouse and human tissues and cells,^19^ and has been applied to various diseases, including astheno‐teratozoospermia and atherosclerosis.^35^, ^36^

Interestingly, our results found that rsRNAs were the most abundant sncRNAs in the atrial tissues of AF, and the proportions of tsRNAs and miRNAs were comparable, which is consistent with the results of PANDORA sequencing and a recent study on sncRNA profiles in acute myeloid leukaemia.^19^, ^37^ Despite being the most abundant sncRNAs, exploration of rsRNAs is still in the initial stages, and their underlying molecular mechanisms remain largely unknown. Previous studies on rsRNA have primarily focused on spermatogenesis, Chu et al. observed a 15‐fold increase in rsRNA‐28S expression in the intestine of mouse 4 days after lipopolysaccharide injection. Additionally, the upper band of rsRNA‐28S was diminished in semen samples from patients with leukocytospermia, indicating a potential involvement of rsRNA in inflammatory processes.^38^ A recent study found that rsRNA‐28S could directly target the 3′ untranslated region of the prostaglandin I2 synthase gene to downregulate its expression and attenuate the chemoresistance of prostate cancer cells.^39^ However, their biological functions in AF or cardiovascular diseases remain unexplored.

Similar to rsRNAs, tsRNAs participate in inflammation responses,^40^ and were dysregulated in the sperm of high‐fat diet mice, suggesting their involvement in mediating the intergenerational inheritance of metabolic disorders induced by diet.^41^ Although different from rsRNA, tsRNA has gained considerable attention; its mechanism of action has been extensively explored, which demonstrates that it can act as miRNAs or piRNAs to participate in gene silencing, regulate chromatin and epigenetic modifications, promote ribosome biogenesis and regulate translation.^15^ Yang et al. examined the tsRNA expression profile using microarray analysis from three pairs of cardiac papillary muscles obtained from patients with rheumatic heart disease with and without AF based on the rtStar tRF & tiRNA Pretreatment Kit (Arraystar, USA) protocols.^13^ Subsequently, the trimmed reads were aligned to mature tRNA or pre‐tRNA sequences using the GtRNAdb. In total, 77 differentially expressed tsRNAs were identified in AF patients.^13^ The qRT‐PCR results validated the differential expression of three tsRNAs, and bioinformatics analysis of their target genes revealed enriched functions related to the regulation of transcription, DNA binding, intracellular, and cytokine–cytokine receptor interactions. Because the patients in this study were different from those in our study and there is no unified nomenclature for tsRNA (tsRNA is named by a certain sequence number in the study by *Yang* et al.^13^), direct comparison of the results from the two studies is not feasible. However, both studies suggested that altered expression of tsRNA may play a regulatory role in the occurrence of AF. Moreover, Xie et al. identified nine differentially expressed tsRNAs in an AF mouse model using high‐throughput sequencing, and mechanistic studies revealed that tsRNA‐5008a promoted myocardial fibrosis by targeting SLC7A11 to regulate ferroptosis.^14^ Further studies are needed to explore the specific mechanism of differentially expressed tsRNAs in AF in the future.

The canonical roles of snoRNAs included involvement in ribosome biogenesis, 2′‐O‐ribose methylation, and pseudouridylation of rRNAs and sncRNAs, and the nonconical roles of snoRNAs included alternative splicing, mRNA 3′ end processing, and miRNA like functions.^42^, ^43^, ^44^ Dysregulated snoRNAs have been implicated in various human diseases.^45^, ^46^ However, their impact on cardiovascular disease remains unclear, and mechanistic studies of snoRNAs in AF are lacking. Håkansson et al. observed varying expression levels of 14q32 snoRNAs throughout the human vasculature, which are highly expressed in the failing heart.^47^ RNA immunoprecipitation experiments suggested that 14q32 snoRNAs can target fibrillarin and AGO1 to exert their effects. This study highlights the important regulatory role of 14q32 snoRNAs in cardiovascular diseases, emphasizing the need for increased attention to snoRNAs in future basic and clinical research on cardiovascular disease. Accordingly, our study identified 51 differentially expressed snoRNAs in AF patients, and their target genes were significantly enriched in the regulation of ion transmembrane transport and MAPK signalling pathways, suggesting that snoRNAs may play regulatory roles in AF pathology.

PiRNAs are a class of sncRNAs that specifically bind to PIWI proteins and form the piRNA silencing complex to regulate gene expression.^48^ No differentially expressed piRNAs in AF were identified in our study, which could be attributed to the overlap of sequences between piRNAs and other sncRNAs. One study compared piRNA databases with other sncRNA databases and found that a substantial number of piRNA‐mapped reads showed 100% identity with other sncRNAs.^49^ Therefore, whether these piRNAs indeed function as piRNAs or belong to other sncRNAs remains to be determined. In our study, SPORTS1.1 software was used for annotation of sncRNAs, which is capable of annotating canonical sncRNAs, such as miRNAs and piRNAs, and is optimized for analysing tsRNAs and rsRNAs.^20^ Because it initially maps reads to miRbase, rRNA database, GtRNAdb and piRNA databases, some piRNAs may be misclassified as miRNAs or other sncRNAs. Currently, mechanistic studies on piRNAs and AF are lacking, warranting further research to investigate whether there are differentially expressed piRNAs in AF and their specific mechanisms.

Many early studies on sncRNAs focused on miRNAs. Cooley et al. measured miRNA expression profiles in the right and left atrial appendage tissues of valvular heart disease patients and found that, compared with patients with sinus rhythm, patients with AF had 15 upregulated miRNAs and 32 downregulated miRNAs in the right atrial tissues, whereas no differentially expressed miRNAs were found in the left atrial tissues.^50^ Among the 34 differentially expressed miRNAs in our study, only two miRNAs (hsa‐miR‐133b and hsa‐146b‐5p) were dysregulated, consistent with the findings of Cooley et al. Different sequencing approaches might have contributed to the identification of a few consistent differentially expressed miRNAs in these studies. Furthermore, Cooley et al. used microarrays and applied a less stringent significance criterion of fold change >1.5. However, although both studies included patients with valvular heart disease, Cooley et al. included older people with coexisting conditions such as diabetes; age and other diseases are associated with dysregulated miRNAs.^51^, ^52^ Moreover, hsa‐146b‐5p was upregulated in the left atrial appendage of patients with non‐valvular paroxysmal AF.^53^ Further mechanistic studies confirmed that hsa‐146b‐5p targeted tissue inhibitor of metalloproteinases 4 and reduced their expression, which in turn activated matrix metalloproteinase‐9 which contributed to increased atrial fibrosis. In addition, the altered expression of miRNAs is involved in the electrical remodelling of AF,^9^ such as miR‐155 targeting CACNA1C to regulate I_Ca,L_,^54^ which is consistent with our results that the target genes of differentially expressed miRNAs were mainly enriched in the ‘calcium signaling pathway.’

This study comprehensively analysed the sncRNA profiles in AF patients using PANDORA‐sequencing and found that rsRNAs were the most abundant sncRNAs, followed by tsRNAs and miRNAs which were originally considered the most abundant. Compared with traditional high‐throughput sequencing, PANDORA‐sequencing overcomes RNA modification‐elicited sequence interferences and qualifies sncRNA profiles more accurately.^19^ Additionally, previous studies on AF‐related sncRNAs have primarily focused on the expression profile of a certain class of sncRNAs. Our study systematically revealed the expression profiles of different classes of sncRNAs in AF and identified various differentially expressed sncRNAs, suggesting that among the sncRNAs, rsRNAs, tsRNAs and miRNAs may be dominant in AF. These findings provide potential insights for further exploration of the mechanisms underlying the onset and progression of AF and the identification of potential biomarkers for AF treatment and prognosis. Given that current research on sncRNAs in AF mainly focuses on miRNAs, more studies are needed to explore the role and mechanism of other classes of sncRNAs in AF.

### 4.1 Limitations

Our study had certain limitations. First, the sample size was relatively small, which might have contributed to bias and false‐negative results owing to insufficient statistical power. However, as an exploratory study, our results identified differentially expressed sncRNAs that may play a role in AF. Notably, one of the differentially expressed tsRNAs was associated with an increased risk of mortality in AF patients, which may lay the foundation for further mechanistic research. Future studies with larger sample sizes are needed to examine the expression profiles of sncRNAs in AF, and the sequencing results should be further validated using qRT‐PCR in larger samples. Second, although bioinformatics analyses predicting target genes of differentially expressed sncRNAs and potential enrichment pathways were provided, the underlying biological mechanisms were not sufficiently detailed, warranting further research on the specific molecular mechanisms of differentially expressed sncRNAs in AF. Third, we examined the association between only one differentially expressed tsRNA and AF prognosis in a relatively small sample using the Cox proportional hazards model. The prognostic role of tsRNA 5006c‐LysCTT in AF needs to be validated in larger prospective cohort studies. Further research is required to explore whether other sncRNAs have potential as biomarkers for AF treatment and prognosis. Furthermore, given the superior performance of many computational models for non‐coding RNA‐disease association prediction, such as machine learning‐based models,^11^, ^12^ these models should be used to evaluate the association between different classes of sncRNAs and AF as more data become available in the future.

## 5 CONCLUSION

This is the first study to comprehensively describe the expression profiles of sncRNAs in the atrial appendages of AF and non‐AF patients with valvular heart disease. Compared with non‐AF patients, a large number of dysregulated sncRNAs were observed in AF patients, and their target genes were mainly enriched in the ‘calcium signaling pathway’ and ‘adrenergic signaling in cardiomyocytes,’ which indicated their potential regulatory roles in AF. Further research is required to explore the specific molecular mechanisms involved in the pathogenesis of AF.

## AUTHOR CONTRIBUTIONS

**Yuhong Zeng:** Data curation (equal); formal analysis (equal); methodology (equal); writing – original draft (lead). **Zhiquan Yuan:** Data curation (equal); writing – original draft (supporting). **Jun Li:** Data curation (equal); methodology (equal); writing – review and editing (supporting). **Lanqing Yang:** Data curation (equal). **Chengying Li:** Data curation (equal). **Ying Xiang:** Data curation (equal). **Long Wu:** Data curation (equal). **Tingting Xia:** Data curation (equal). **Li Zhong:** Conceptualization (equal); supervision (equal); writing – review and editing (equal). **Yafei Li:** Conceptualization (equal); funding acquisition (equal); supervision (equal); writing – review and editing (equal). **Na Wu:** Conceptualization (equal); funding acquisition (lead); supervision (equal); writing – review and editing (equal).

## FUNDING INFORMATION

This study was supported by the National Natural Science Foundation of China (No. 82073649 to N.W.).

## CONFLICT OF INTEREST STATEMENT

The authors have no conflict of interest to declare.

## PATIENT CONSENT

Written informed consent was obtained from each enrolled participant.

## Supporting information


Data S1.

## Data Availability

The original contributions presented in the study are included in the article/Supplementary files, further inquiries can be directed to the corresponding author/s.
