# Supplementary material for: Small non‐coding RNA signatures in atrial appendages of patients with atrial fibrillation
Source: J Cell Mol Med. 2024 Jun 22;28(12):e18483. doi: 10.1111/jcmm.18483 (PMC11193094; doi:10.1111/jcmm.18483)
Supplement: Supplementary file 1 — Data S1. [file JCMM-28-e18483-s001.docx]

**Supplemental Materials**

# Supplemental tables

**Supplementary Table 1** Primer sequences used in the study.

| **Name** | **Primer Sequences (5'- 3')** |
| --- | --- |
| tsRNA-5032b-AspGTC | Stem-Loop: GTCGTATCCAGTGCAGGGTCCGAGGTATTCGCACTGGATACGACACTCAC  Forward: GCGCTCCTCGTTAGTATAGTG |
| tsRNA-5006c-LysCTT | Stem-Loop: GTCGTATCCAGTGCAGGGTCCGAGGTATTCGCACTGGATACGACGTCCCA  Forward: AAGAATGCCCGGCTAGCTCA |
| tsRNA-5030c-GluCTC | Stem-Loop: GTCGTATCCAGTGCAGGGTCCGAGGTATTCGCACTGGATACGACCGCCGA  Forward: AAGAGCGTTCCCTGGTGGTCT |
| Universal Reverse | ATCCAGTGCAGGGTCCGAGG |
| SNORA44 | Forward: CAGCATGTTTCCAAGGGCTG  Reverse: GTGGCAGCTTGCAGGTATTG |
| SNORD55 | Forward: CGGTAATGCTGCATACTCCCGA  Reverse: AGCTCTCCAAGGTTGGCTTCC |
| SNORA72 | Forward: GTCAGGACAGGCTAAACATTCG  Reverse: AAGAACTAGGTTTGGGGACACA |
| U6 | Forward: GCTTCGGCAGCACATATACTAAAAT  Reverse: CGCTTCACGAATTTGCGTGTCAT |

**Supplementary Table 2** Top 10 upregulated and downregulated differentially expressed sequences of rsRNAs in AF patients compared with non-AF patients.

| seq_annotation | Expression | Log_2_FoldChage | P value |
| --- | --- | --- | --- |
| ACAGCTAAAAGAGCACACCCGTCTATGTAGCAAAATAGTG, 16S-rRNA | Up | 4.73 | 0.032 |
| TAGTTGAACAGGGCCCTGAAGCGCGTACACACCGCCCGTCACC, 12S-rRNA | Up | 4.50 | 0.015 |
| CGCGTCCCCCGAAGAGGGGGACGGCGGAGCGAGCGCACGGGGT, 28S-rRNA | Up | 4.46 | 0.044 |
| CATAGTAGGCCTAAAAGCAGCCACCAATTAAGAAAGCGTTCA, 16S-rRNA | Up | 4.44 | 0.039 |
| GCAGTTTTATCCGGTAAAGCGAATGATTAGAGGTC, 28S-rRNA | Up | 4.24 | 0.009 |
| TATCAACAATAGGGTTTACGACCTCGATGTTGGATCAGG, 16S-rRNA | Up | 4.17 | 0.029 |
| GAATGGCTCATTAAATCAGTTATGGTTCCTTTGGTCG, 18S-rRNA | Up | 4.03 | 0.011 |
| ACTCCATCTAAGGCTAAATACCGGCACGAGACCGA, 28S-rRNA | Up | 4.01 | 0.049 |
| TTTGGTCCTAGCCTTTCTATTAGCTCTTAGTAAG,12S-rRNA | Up | 3.99 | 0.027 |
| TTTGCCAAGAATGTTTTCATTAATCAAGA,  18S-rRNA | Up | 3.94 | 0.010 |
| ACGCCGGCAGGCGCGGGTAACCCGTTGAACCCCATTCGT, 18S-rRNA | Down | -4.58 | 0.022 |
| ATCCGGGGGAGAGGGTGTAAATCTCGCGC,  28S-rRNA | Down | -4.45 | 0.028 |
| CACGTCTCGTCGCGCGCGCGTCCGCTGGGGGC,  28S-rRNA | Down | -4.34 | 0.018 |
| TCAGACATTTGGTGTATGTGCTTGGCTGAGGAGCCAATGGGGC, 28S-rRNA | Down | -4.31 | 0.041 |
| TCCCACTGTCCCTACCTACTATCCAGCGAA,  28S-rRNA | Down | -4.27 | 0.004 |
| CACTACGAGCCACAGCTTAAAACTCA, 12S-rRNA | Down | -4.22 | 0.001 |
| CGCCGGGCAGCTTCCGGGAAACCA, 18S-rRNA | Down | -4.03 | 0.007 |
| GAAGGCCCGCGGCGGGTGTTGACGCGATGT,  28S-rRNA | Down | -4.01 | 0.024 |
| GAGGTAAACGGGTGGGGTCCGCGCAGTCCGCCCGGAGGATTC, 28S-rRNA | Down | -3.99 | 0.045 |
| TCAAACGGTAACGCAGGTGTCCTAAGGCGAGCTCAGGGAGGAC, 28S-rRNA | Down | -3.98 | 0.038 |

**Supplementary Table 3** Top 10 upregulated and downregulated differentially expressed of miRNAs in AF patients compared with non-AF patients.

| miRNAs | Expression | Log_2_FoldChage | P value |
| --- | --- | --- | --- |
| hsa-miR-217-5p | Up | 3.44 | 0.006 |
| hsa-miR-146b-3p | Up | 3.02 | 0.040 |
| hsa-miR-208b-3p | Up | 2.59 | 0.035 |
| hsa-miR-221-3p | Up | 2.43 | 0.032 |
| hsa-miR-24-3p | Up | 2.17 | 0.025 |
| hsa-miR-146b-5p | Up | 1.60 | 0.002 |
| hsa-miR-187-3p | Up | 1.59 | 0.001 |
| hsa-miR-30b-5p | Up | 1.33 | 0.037 |
| hsa-miR-212-5p | Up | 1.22 | 0.026 |
| hsa-miR-224-5p | Up | 1.05 | 0.011 |
| hsa-miR-196b-5p | Down | -3.94 | 0.048 |
| hsa-miR-205-5p | Down | -3.33 | 0.027 |
| hsa-miR-382-5p | Down | -3.12 | 0.016 |
| hsa-miR-3651 | Down | -3.00 | 0.024 |
| hsa-miR-323a-3p | Down | -2.81 | 0.046 |
| hsa-miR-26a-5p | Down | -2.42 | 0.047 |
| hsa-miR-200b-3p | Down | -2.37 | 0.031 |
| hsa-let-7b-5p | Down | -2.30 | 0.031 |
| hsa-miR-15b-3p | Down | -2.29 | 0.011 |
| hsa-miR-200a-3p | Down | -2.07 | 0.005 |

**Supplementary Table 4** Top 10 upregulated and downregulated differentially expressed sequences of tsRNAs in AF patients compared with non-AF patients.

| seq_annotation | Expression | Log_2_FoldChage | P value |
| --- | --- | --- | --- |
| AAAACATCAGATTGTGAATCTGACAACAGAGGCTTACGACCC, mature-mt_tRNA-His-GTG | Up | 5.33 | 0.033 |
| TTGTGAATCTGACAACAGAGGCTTACGACCCCTTATTTACCC, mature-mt_tRNA-His-GTG | Up | 5.28 | 0.009 |
| AGTTTAACCAAAACATCAGATTGTGAATCTGACAACAGAGGCTT, mature-mt_tRNA-His-GTG | Up | 5.20 | 0.009 |
| AGATTGTGAATCTGACAACAGAGGCTTACGACCCCTTAT, mature-mt_tRNA-His-GTG | Up | 4.58 | 0.024 |
| AAAACATCAGATTGTGAATCTGACAACAGAGGCTC, mature-mt_tRNA-His-GTG | Up | 4.50 | 0.044 |
| CTGAAGGTCCTGAGTTCGAACCTCAGAGGGGGCACC, mature-tRNA-Met-CAT | Up | 4.44 | 0.019 |
| ATCACATTCGCCTCACACGCGAAAGGTCCCCGGT,mature-tRNA-Val-CAC | Up | 4.36 | 0.049 |
| CTCACAAGAACTGCTAACTCATGCCCCCATGTCTAACA, mature-mt_tRNA-Ser-GCT | Up | 4.31 | 0.014 |
| TGAGGGTCCAGGGTTCAAGTCCCTGTTCGG, mature-tRNA-Lys-TTT | Up | 4.04 | 0.003 |
| AACTGCTAACTCATGCCCCCATGTCTAACAACA, mature-mt_tRNA-Ser-GCT | Up | 3.99 | 0.011 |
| TTCAGTGGTAGAATTCTCGCCTGCCACGCGGGAGGCCCGGG, mature-tRNA-Gly-GCC | Down | -4.97 | 0.013 |
| ATTCCTGGTTTTCACCCAGGCGGCCCG,  mature-tRNA-Glu-TTC | Down | -4.39 | 0.005 |
| TAGCGGTTAGGATTCCTGGTTTTCACCCAGGCGGCC, mature-tRNA-Glu-TTC | Down | -4.24 | 0.019 |
| GTGGTAGAATTCTCGCCTGCCACGCGGGAGGCCCGGGTTC, mature-tRNA-Gly-GCC | Down | -4.21 | 0.049 |
| ACTTAAAACTTTACAGTCAGA,  mature-mt_tRNA-Leu-TAA | Down | -4.13 | 0.040 |
| AGTGGTTAGTATCCCCGCCTGT,  mature-tRNA-Asp-GTC | Down | -4.02 | 0.020 |
| CCCAGGTGGCCCGGG, mature-tRNA-Glu-TTC | Down | -3.90 | 0.042 |
| CAACTTACACTTAGGAGATTTCAACTTAACTTGACCGCTCTGAC, mature-mt_tRNA-Val-TAC | Down | -3.81 | 0.040 |
| TTGCGACCCGGGTTC, mature-tRNA-Gly-CCC | Down | -3.74 | 0.048 |
| GGATTCCTGGTTTTCACCCAGGCGGCC,  mature-tRNA-Glu-TTC | Down | -3.65 | 0.028 |

**Supplementary Table 5** Top 10 upregulated and downregulated differentially expressed of snoRNAs in AF patients compared with non-AF patients.

| SnoRNAs | Expression | Log_2_FoldChage | P value |
| --- | --- | --- | --- |
| SNORD95 | Up | 4.32 | 0.023 |
| SNORD57 | Up | 4.02 | 0.034 |
| SNORD26 | Up | 3.79 | 0.018 |
| SNORD6 | Up | 3.79 | 0.045 |
| SNORD48 | Up | 3.14 | 0.039 |
| SNORD114-21 | Up | 3.10 | 0.044 |
| SNORD99 | Up | 3.03 | 0.049 |
| SNORD116-27 | Up | 2.89 | 0.034 |
| SNORD51 | Up | 2.85 | 0.026 |
| SNORD43 | Up | 2.84 | 0.018 |
| SNORA44 | Down | -4.71 | 0.005 |
| SNORD61 | Down | -3.88 | 0.006 |
| SNORD69 | Down | -3.87 | 0.044 |
| SNORD30 | Down | -3.84 | 0.020 |
| SNORA54 | Down | -3.79 | 0.049 |
| SNORA46 | Down | -3.62 | 0.038 |
| SNORA36A | Down | -3.60 | 0.041 |
| SNORA20 | Down | -3.55 | 0.042 |
| SNORA80E | Down | -3.28 | 0.022 |
| SNORD25 | Down | -3.24 | 0.027 |

**Supplementary Table 6** Clinical characteristics of 127 patients with AF.

| characteristics | N（n=127） |
| --- | --- |
| Age | 67.7±11.1 |
| <65y | 41 (32.3) |
| 65-74y | 51 (40.2) |
| ≥75y | 35 (27.6) |
| Male | 67 (52.8) |
| AF type |  |
| Paroxysmal AF | 59 (46.5) |
| Persistent or permanent AF | 68 (53.5) |
| BMI | 24.1±3.5 |
| <18.5 | 58 (45.7) |
| 18.5-23.9 | 6 (4.7) |
| ≥24 | 63 (49.6) |
| Education level |  |
| ≤Junior high school | 113 (89.0) |
| ≥High school | 14 (11.0) |
| Smoking | 45 (35.4) |
| Drinking | 32 (25.2) |
| Comorbidities |  |
| Hypertension | 72 (56.7) |
| Diabetes | 21 (16.5) |
| Hyperlipidemia | 29 (22.8) |
| Coronary heart disease | 59 (46.5) |
| Heart failure | 45 (35.4) |
| Stroke/TIA | 22 (17.3) |
| Peripheral vascular disease | 5 (3.9) |
| Left atrial diameter (mm） | 45.2±8.7 |
| LVEF (%) | 55.5±11.4 |
| Drug therapy |  |
| ACEI/ARB | 38 (29.9) |
| β-blocker | 37 (29.1) |
| Calcium channel blocker | 37 (29.1) |
| Warfarin | 10 (7.9) |
| Aspirin | 20 (15.7) |
| Clopidogrel | 20 (15.7) |
| Statins | 36 (28.3) |
| CHA2DS2-VASc score | 3 (1.5, 4) |

AF, atrial fibrillation; BMI, body mass index; TIA, transient ischemic attack; ACEI, angiotensin-converting enzyme inhibitors; ARB, angiotensin receptor blocker; LVEF, left ventricular ejection fraction.

# Supplemental figures with accompanying figure legends

**Supplementary Figure Legends**

Supplementary Figure 1. Flowchart of data collection and method implementation.

Supplementary Figure 2. Go analysis for the target genes of differentially expressed (A) miRNAs, (B) snoRNAs (C) nucleus-encoded tsRNAs and (D) mitochondria-encoded tsRNAs. The miRNA indicated for microRNA; snoRNA indicated for small nucleolar RNA; tsRNA indicated for transfer RNA-derived small RNA.


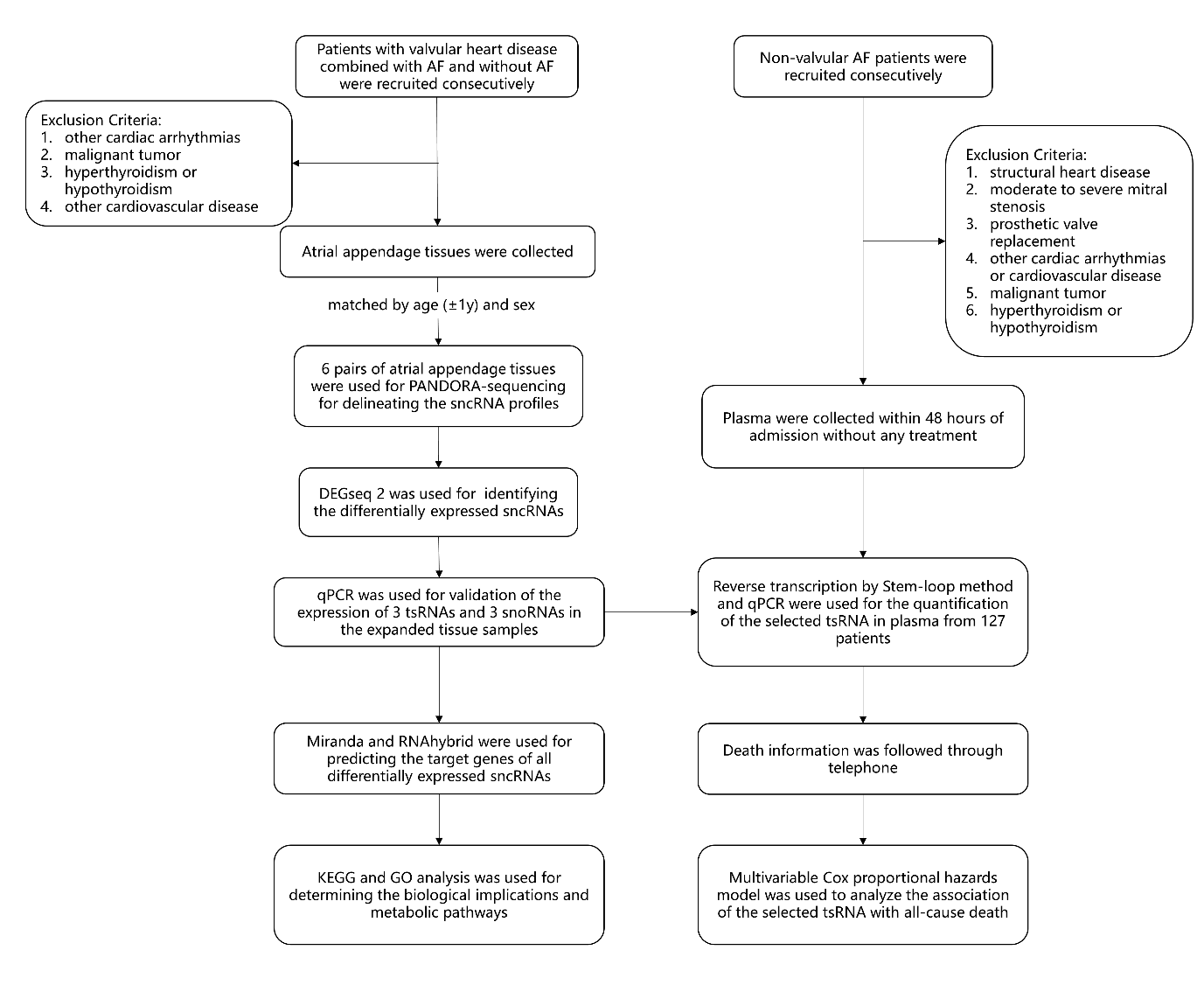


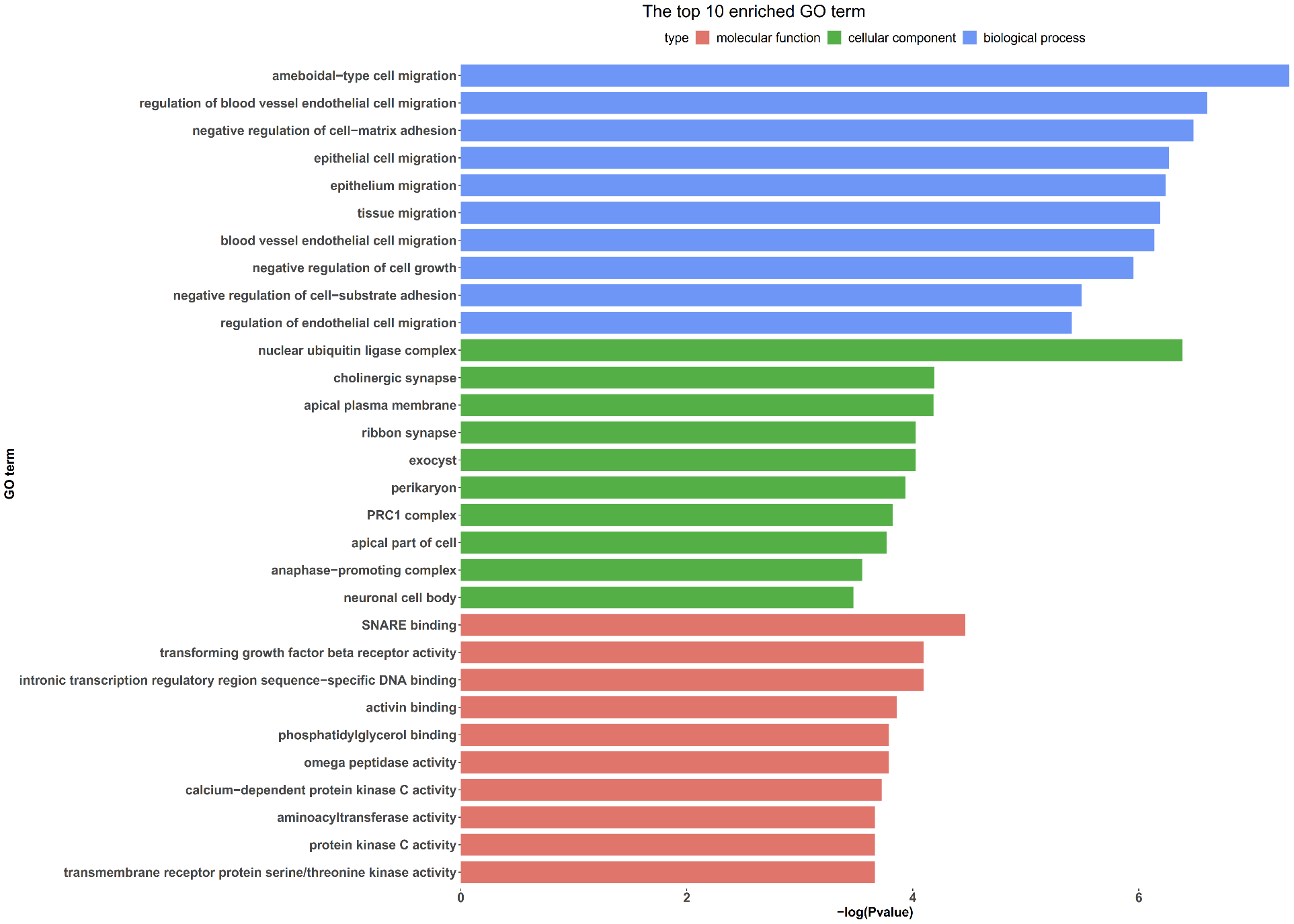


1. miRNA


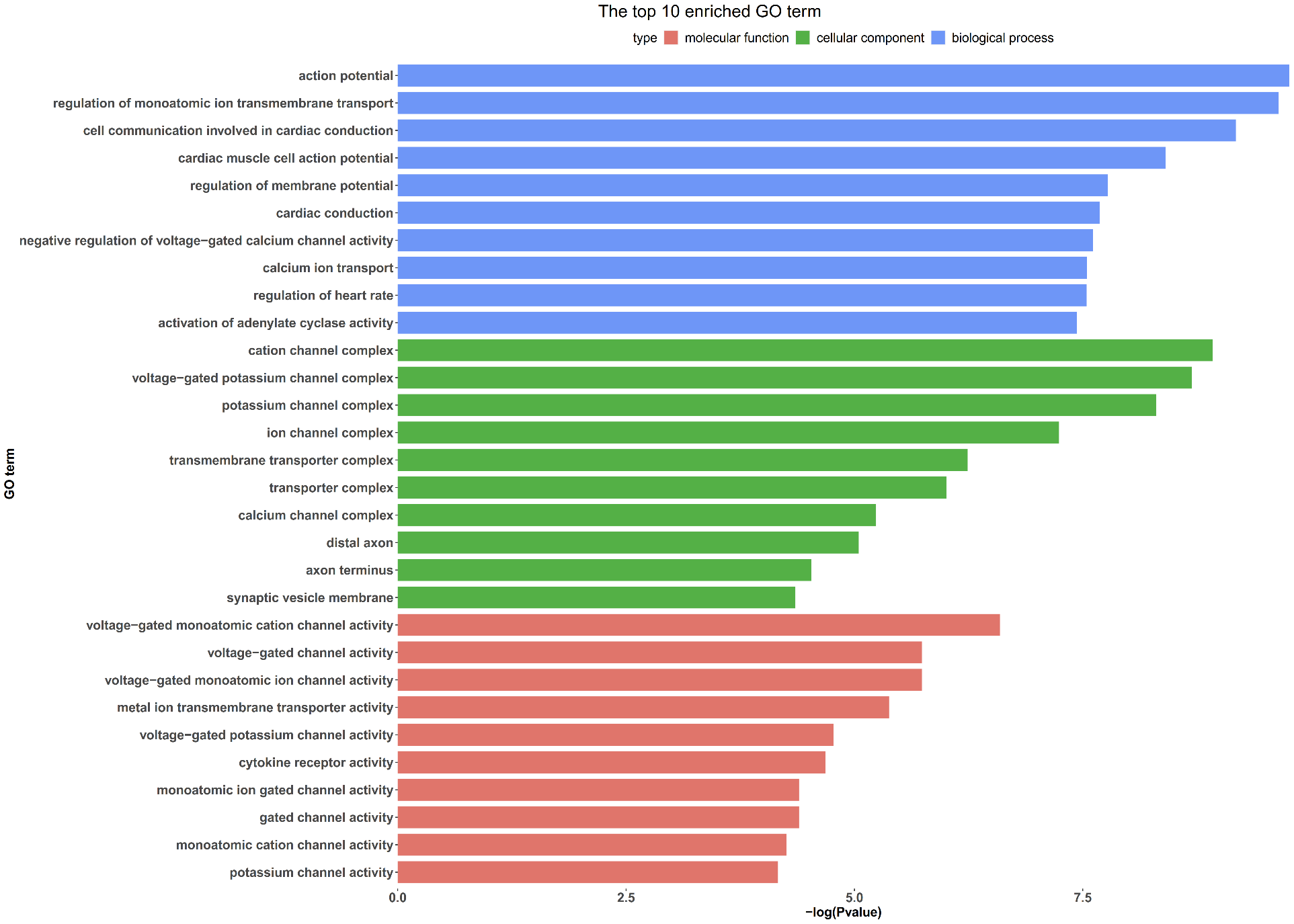


1. snoRNAs


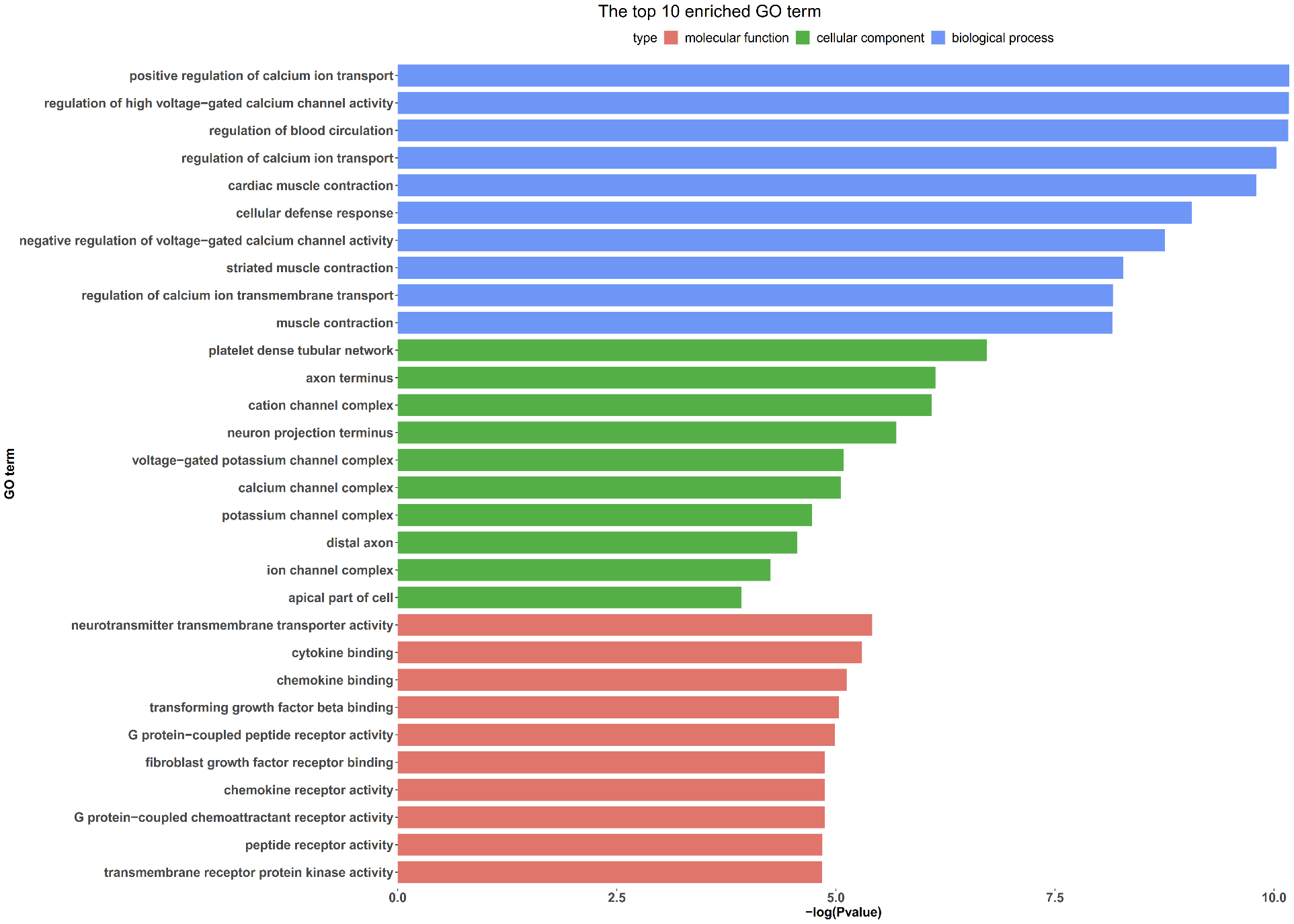


1. nucleus-encoded tsRNAs


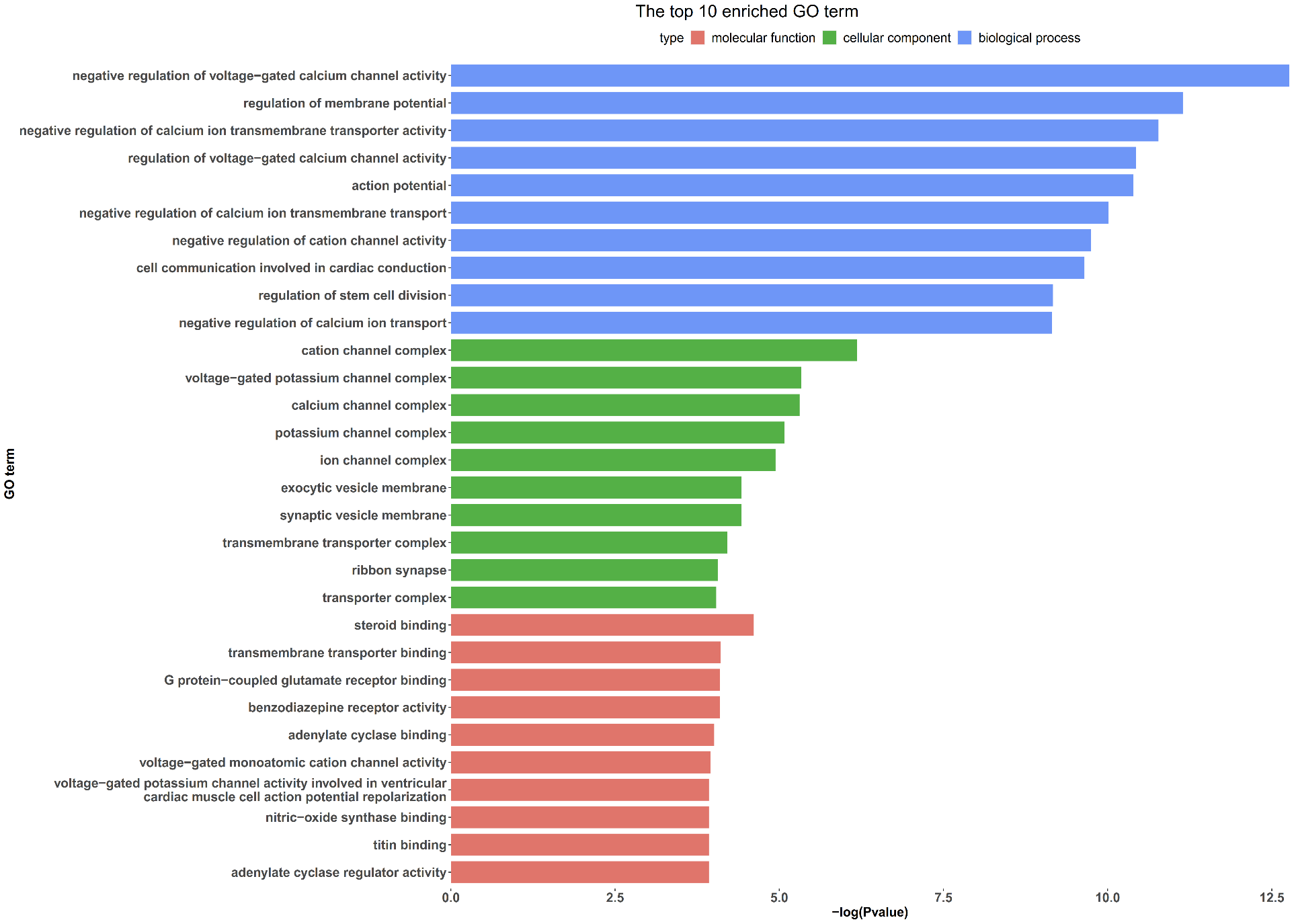


1. mitochondria-encoded tsRNAs
